# Exploring potential strategies for haploid induction based on double fertilization in plants

**DOI:** 10.1111/pbi.70197

**Published:** 2025-06-18

**Authors:** Tengyu Li, Chenlei Wang, Jingwen Pan, Javaria Tabusam, Yan Li, Jinbo Yao, Wei Chen, Yazhong Wang, Wei Gao, Junkang Rong, Zeeshan Ahmad, Andreas Houben, Shouhong Zhu, Shuangxia Jin, Yongshan Zhang

**Affiliations:** ^1^ State Key Laboratory of Cotton Bio‐breeding and Integrated Utilization, Institute of Cotton Research Chinese Academy of Agricultural Science Anyang China; ^2^ National Key Laboratory of Crop Genetic Improvement Huazhong Agricultural University Wuhan China; ^3^ Department of Breeding Research Leibniz Institute of Plant Genetics and Crop Plant Research (IPK) Gatersleben Seeland Germany; ^4^ Department of Chromosome Biology Max Planck Institute for Plant Breeding Research Cologne Germany; ^5^ National Key Laboratory of Cotton Bio‐breeding and Integrated Utilization Henan University Henan China; ^6^ The Key Laboratory for Quality Improvement of Agricultural Products of Zhejiang Province Zhejiang Agricultural and Forestry University Hangzhou China

**Keywords:** haploid induction, double fertilization, sperm DNA fragmentation, membrane fusion, uniparental chromosome elimination, ectopic expression

## Abstract

Haploid induction (HI), an indispensable procedure in doubled haploid breeding, has attracted increasing attention in crop genetic improvement due to its ability to rapidly fix desirable traits in a homozygous state, thereby shortening the breeding cycle. However, HI has only been successfully implemented in a limited number of crops, and its underlying mechanisms remain largely enigmatic. This review summarizes five potential HI routes based on previous findings and the key events during the process of double fertilization in flowering plants. Among these HI methods, we suggest that sperm DNA fragmentation and ectopic expression of embryogenesis activator, as straightforward avenues for discovering new HI‐related genes. We also emphasize that the combination of genome editing techniques with HI is a promising strategy to accelerate crop improvement and doubled haploid breeding. We envision that the proposed directions can pave the way for improving and deepening our understanding of HI mechanisms.

## Introduction

Haploid induction (HI), a critical step in doubled haploid technology, accelerates the generation of homozygous lines compared with the long conventional breeding cycle and promotes the high‐speed development of new cultivars (Dunwell, 2010; Dwivedi *et al*., 2015). Traditional methods for HI mainly rely on *in vitro* regeneration of haploid gametophytic cells within cultivated explants, such as pistils, ovules, anthers or isolated immature pollen (Gao *et al*., 2024; Gurel *et al*., 2021). Alternatively, *in vivo* haploid induction, such as distant hybridization and haploid inducer genotypes, is being used (Forster *et al*., 2007; Ishii *et al*., 2016). Haploidy may arise following pollination with pollen exposed to physical stress (radiation and high temperature) or chemical treatments (phosphatidylcholine or methimazole) (Jiang *et al*., 2022; Katayama, 1934; Mathur *et al*., 1980; Shen *et al*., 2023). However, the wide application of these approaches has been limited by various factors such as the availability of inducer genotype, recalcitrant species and genotype bottleneck regarding regeneration.

**Figure 1:**
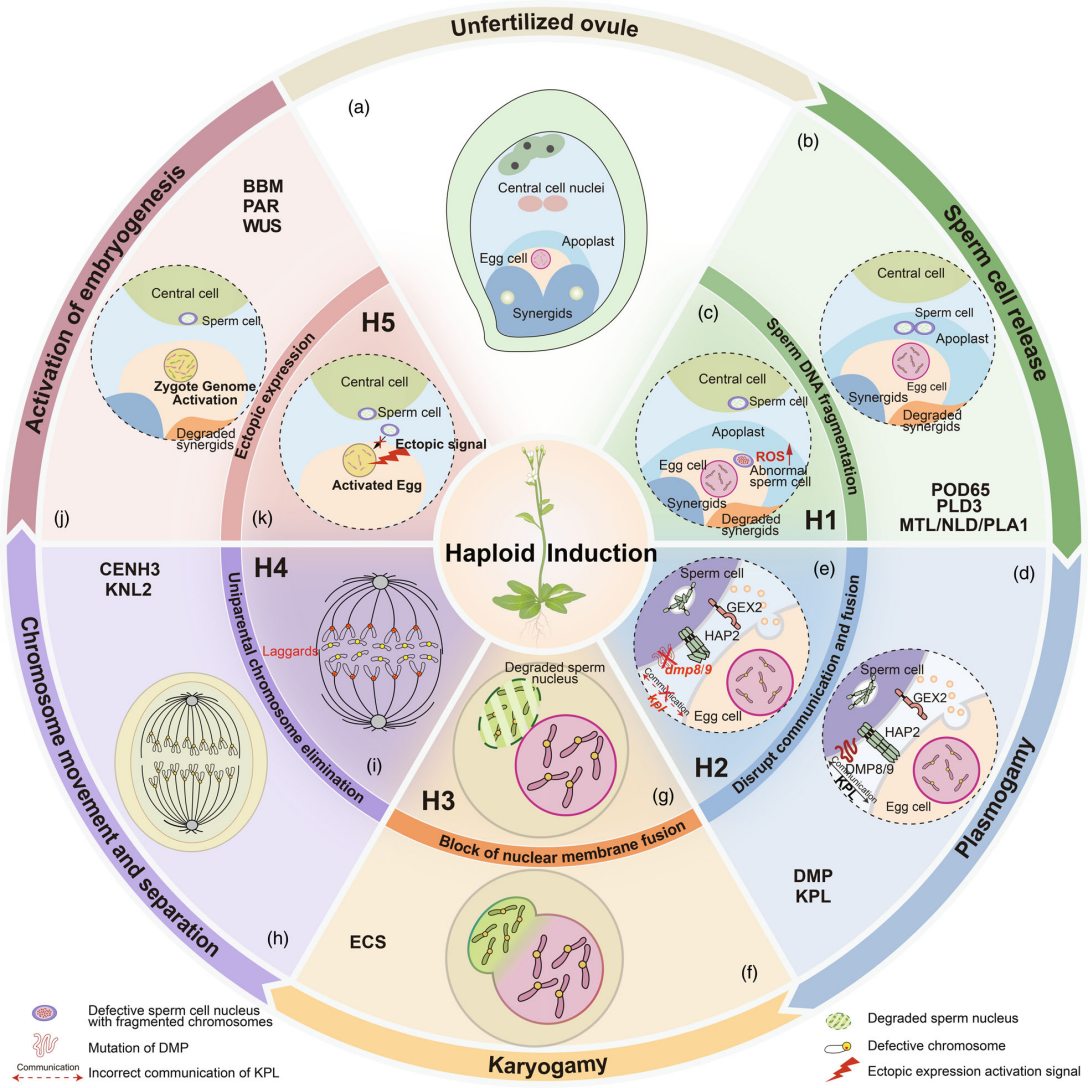
Hypothetical mechanistic model of HI according to double fertilization in plants. (a) Diagram of an unfertilized ovule showing its eight constituent nuclei. (b) During the first stage of double fertilization, after the pollen tube ruptures, sperm cells are released and enter the embryo sac. (c) First method for HI (H1): Elevated reactive oxygen species (ROS) levels result in the fragmentation of sperm DNA and trigger HI. (d) Communication and fusion between the plasma membranes of the male and female gametes. (e) Second method for HI (H2): A haploid state is induced by disrupting plasma membrane communication (requiring *KOKOPELLI* [*KPL*]) or fusion (requiring *DOMAIN OF UNKNOWN FUNCTION 679 PROTEIN* [*DMP*]). (f) Fusion of the nuclear membranes from the sperm cell and the egg cell. (g) Third method for HI (H3): Blocking the fusion of the nuclear membranes results in the degradation of the sperm nucleus. (h) Chromosome movement and separation before initiation of zygote development. (i) Fourth method for HI (H4): CENTROMERIC HISTONE H3 (CENH3) function is directly (with *cenh3* mutants) or indirectly (with mutation of *KINETOCHORE NULL 2* [*KNL2*]) disturbed, causing the elimination of chromosomes from the maternal or paternal genome during early embryogenesis. (j) Activation of embryogenesis. (k) Fifth method for HI (H5): Ectopic expression of BABY BOOM (*BBM*), *PARTHENOGENESIS* (*PAR*), or *WUSCHEL* (*WUS*) leads to advanced activation of egg cells to generate haploids with only the maternal genome.

Double fertilization is the defining feature of flowering plants and involves the fusion of two male gametes with two separate female gametes, forming a diploid zygote and a triploid endosperm (Dresselhaus *et al*., 2016; Sprunck, 2020). Successful double fertilization is a complex process and requires numerous cell‐to‐cell communication events between gametophytes. The known HI genes are involved in either one of the following processes of double fertilization (Figure 1): (1) maintaining sperm cell integrity, (2) plasmogamy (fusion of the plasma membranes from the egg and sperm cells), (3) karyogamy (fusion of the nuclear membranes), (4) regulation of cell division and (5) activation of embryogenesis (Anderson *et al*., 2017; Johnson *et al*., 2019; Zhao *et al*., 2022a). This approach is based on interfering with the normal course of double fertilization in angiosperms to produce haploid plants with only one set of parental chromosomes instead of the usual two (Gilles *et al*., 2017b).

*In vivo* haploid induction is an innovative approach which plays an increasingly important role in accelerated crop improvement (Table 1). However, the production of haploids is a rare and specialized event during double fertilization. A limited number of genes have been harnessed for HI, and the molecular mechanisms underlying their function of some genes are still unknown. Understanding the molecular mechanism behind the haploid induction process enables the widespread application of HI technology. In this review, we attempt to classify the previously reported genes responsible for haploid induction based on their involvement in five critical events in the double fertilization process. The importance of the HI gene to their corresponding function related to fertilization and the reason for haploid induction is described further. Integrating the roles of known HI genes in double fertilization would be helpful to uncover new genes that are involved in those events, which could be potentially used for haploid induction.

**Table 1:** List of *in vivo* HI system according to the double fertilization process in various plant species

Stage of double fertilization	Method of induction	Gene	Type of induction	Species	References
Sperm cell release	H1 Sperm DNA fragmentation	MTL/PLA/NLD	CRISPR/Cas9	*Zea mays*	(Gilles *et al*., 2017a; Kelliher *et al*., 2017, 2019; Liu *et al*., 2017)
			TILLING	*Oryza sativa*	(Yao *et al*., 2018)
			CRISPR/Cas9 CRISPR/SpCas9	*Triticum aestivum*	(Liu *et al*., 2020a, 2020b; Sun *et al*., 2022)
			CRISPR/Cas9	*Setaria italica*	(Cheng *et al*., 2021)
			CRISPR/Cas9/RNAi	*Arabidopsis thaliana*	(Jang *et al*., 2023)
			CRISPR/zCas9	*Hordeum vulgare*	(Tang *et al*., 2023)
			CRISPR/Cas9	*Saccharum* spp.	(Guo *et al*., 2024)
		POD65	CRISPR/Cas9	*Zea mays*	(Jiang *et al*., 2022)
		PLD3	CRISPR/Cas9	*Zea mays*	(Li *et al*., 2021)
Plasma membrane fusion	H2 Disruption of plasma membrane communication and fusion	DMP	CRISPR/Cas9	*Zea mays*	(Zhong *et al*., 2019)
			CRISPR/Cas9	*Arabidopsis thaliana*	(Zhong *et al*., 2020)
			CRISPR/Cas9	*Citrullus lanatus*	(Chen *et al*., 2023; Tian *et al*., 2023)
			CRISPR/Cas9	*Brassica napus*	(Li *et al*., 2022; Zhong *et al*., 2022b)
			CRISPR/Cas9	*Solanum tuberosum* L.	(Zhang *et al*., 2022)
			CRISPR/Cas9	*Brassica oleracea*	(Zhao *et al*., 2022b)
			CRISPR/Cas9	*Nicotiana tabacum*	(Zhong *et al*., 2022b)
			CRISPR/Cas9	*Solanum lycopersicum*	(Zhong *et al*., 2022a)
			CRISPR/Cas9	*Gossypium hirsutum*	(Long *et al*., 2023)
			CRISPR/Cas9	*Medicago truncatula*	(Wang *et al*., 2022b)
			CRISPR/Cas9	*Glycine max*	(Zhong *et al*., 2024)
		KPL	T‐DNA insertion	*Arabidopsis thaliana*	(Jacquier *et al*., 2023)
Nuclear membrane fusion	H3 Blocking nuclear membrane fusion	ECS1/ECS2	T‐DNA insertion	*Arabidopsis thaliana*	(Mao *et al*., 2023; Zhang *et al*., 2023)
			CRISPR/Cas9	*Oryza sativa*	(Zhang *et al*., 2023)
Chromosome movement and separation	H4 Uniparental chromosome elimination	CENH3	GFP‐tailswap	*Arabidopsis thaliana*	(Ravi *et al*., 2014; Ravi and Chan, 2010)
			CRISPR/Cas9/ EMS‐mutagenesis	*Arabidopsis thaliana*	(Kelliher *et al*., 2019; Kuppu *et al*., 2020)
			Point mutation	*Arabidopsis thaliana*	(Karimi‐Ashtiyani *et al*., 2015; Kuppu *et al*., 2020, 2015)
			BnCENH3; LoCENH3; ZmCENH3	*Arabidopsis thaliana*	(Maheshwari *et al*., 2015)
			GFP‐tailswap	*Zea mays*	(Kelliher *et al*., 2016)
			CRISPR/Cas9	*Zea mays*	(Kelliher *et al*., 2019; Wang *et al*., 2021)
			Point mutation	*Solanum lycopersicum*	(Rik *et al*., 2017)
			Point mutation	*Oryza sativa*	(Lv and Kelliher, 2023)
			Point mutation	*Cucumis melo* L.	(Maria *et al*., 2017)
			CRISPR/Cas9	*Triticum aestivum*	(Lv *et al*., 2020)
			RNAi	*Allium cepa* L.	(Manape *et al*., 2024)
			CRISPR/Cas9 Point mutation	*Brassica oleracea*	(Han *et al*., 2024; Wang *et al*., 2024a)
		KNL2	T‐DNA insertion	*Arabidopsis thaliana*	(Ahmadli *et al*., 2023)
Activation of embryogenesis	H5 Ectopic expression of the embryogenesis activator	BBM	PsASGR‐BBML	*Pennisetum squamulatum*	(Conner *et al*., 2015)
			PsASGR‐BBML	*Zea mays*	(Conner *et al*., 2017)
			EC1.2pro:: ZmBBM1	*Zea mays*	(Skinner *et al*., 2023)
			PsASGR‐BBML	*Nicotiana tabacum*	(Zhang *et al*., 2020)
			PsASGR‐BBML	*Oryza sativa*	(Conner *et al*., 2017)
			pDD45::SiBBM1‐3	*Oryza sativa*	(Chahal *et al*., 2022)
			pDD45::OsBBM	*Oryza sativa*	(Khanday *et al*., 2019)
			35S::BnBBM	*Ceratopteris richardii*	(Bui *et al*., 2017)
			pDD45::BnBBM	*Arabidopsis thaliana*	(Chen *et al*., 2022)
			pDD45::BnBBM	*Brassica napus*	(Chen *et al*., 2022)
			pDD45::BnBBM	*Solanum lycopersicon*	(Chen *et al*., 2022)
		PAR	pEC1::ToPAR	*Lactuca sativa*/ *Taraxacum*	(Underwood *et al*., 2022)
			pDD45::ToPAR	*Setaria italica*	(Huang *et al*., 2024)
		WUS	pDD45::OsWUS	*Oryza sativa*	(Huang *et al*., 2025)

## Haploid induction via sperm DNA fragmentation (H1)

The initiation of double fertilization is marked by the release of two sperm cells from the pollen tube into the embryo sac in a position proximal to the egg and the central cell (Figure 1B) (Hamamura *et al*., 2012; Johnson *et al*., 2019). The integrity of sperm nuclei is important to pass the paternal genetic information to the offspring, and the elevated levels of sperm DNA fragmentation result in abnormal embryonic development and pregnancy loss in humans (Marinaro, 2023; Sladjana *et al*., 2021). A study of maize (*Zea mays*) haploid inducer male line revealed that haploid induction is likely caused by fragmentation of sperm DNA during pollen development, as evidenced by sequencing of single sperm nuclei (Li *et al*., 2017).

*MATRILINEAL* (*MTL*), also known as *NOT LIKE DAD* (*NLD*) and *phospholipase A* (*PLA1*), was shown to be the gene underlying the major QTL responsible for haploid induction in *stock 6* inducer in maize (Figure 1B and Figure 1C) (Gilles *et al*., 2017a; Kelliher *et al*., 2017; Liu *et al*., 2017). *M TL*/*NLD*/*PLA1* encodes a phospholipase A protein specifically expressed in pollen (tricellular pollen grains) (Kelliher *et al*., 2017; Liu *et al*., 2017). Transcriptome sequencing (RNA‐seq) analysis showed that the HI ability of the *stock6* line was related to Ca^2+^‐based signalling pathways, including pollen tube guidance and fertilization (Kelliher *et al*., 2017). MTL/NLD/PLA1 is located in the endo‐plasma membrane (endo‐PM, recently redefined as ‘peri‐germ cell membrane’) surrounding sperm cells, but it is absent from the sperm plasma membrane (Gilles *et al*., 2021; Sugi *et al*., 2024). The attachment of this protein to the peri‐germ cell membrane was found to be facilitated by lipid anchoring combined with electrostatic interactions (Gilles *et al*., 2021).

**Figure 2:**
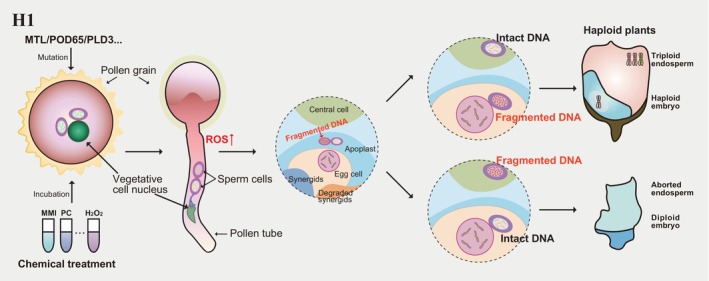
Haploid induction via sperm DNA fragmentation and elevated ROS levels. The mutation of genes related to ROS levels (for example, *MTL*/*NLD*/*PLA1*, *POD65*, or *PLD3*) or treatment of pollen grains with ROS‐inducing reagents (like phosphatidylcholine [PC] and methimazole [MMI]) induces DNA fragmentation in sperm cells and triggers haploid induction.

The discovery that loss‐of‐function mutations of *MTL/NLD/PLA1* trigger HI has led to the investigation of the underlying molecular mechanisms. Possible hypotheses include parthenogenesis (single fertilization) and uniparental chromosome elimination (double fertilization) and have received some experimental support (Chalyk *et al*., 2003; Zhao *et al*., 2013). A hypothesis for single fertilization or double fertilization in HI has been proposed as follows: 1) the mutation of the *MTL/NLD/PLA1* gene leads to chromosome fragmentation of sperm cells; 2) these abnormal sperm cells combine with egg cells to form zygotes after double fertilization; 3) the broken sperm‐derived DNA is gradually lost during zygote development; and 4) haploids are formed (Figure 2) (Jacquier *et al*., 2020).

Reactive oxygen species (ROS) play a prominent role during pollen development, while increased ROS levels are a crucial reason for chromosome fragmentation of sperm DNA (Hu *et al*., 2011; Jiang *et al*., 2022; Sankaranarayanan *et al*., 2020; Yi *et al*., 2016). Integrated multi‐omics data comprising transcriptome, metabolome, quantitative proteome and protein modification data of the *Zmpla1* mutant and wild‐type anthers have illuminated the progression of HI: 1) inactivation of ZmPLA1 protein in triple nucleus pollen leads to the accumulation of phosphatidylcholine; 2) disruption of mitochondrial homeostasis leads to ROS burst; and 3) oxidative damage to chromosomal DNA results in chromosome fragmentation (Jiang *et al*., 2022). This above multi‐omics analysis helped identify the peroxidase gene *ZmPOD65*, which is specifically expressed in the pollen at the three‐nuclei stage and whose loss‐of‐function mutation yielded a haploid induction rate (HIR) of up to 7.7% (Jiang *et al*., 2022). Likewise, the mutation of *ZmPLD3*, a member of the *phospholipase D* subfamily, can induce maternal haploids in maize (Li *et al*., 2021). Knocking out both *ZmPLD3* and *MTL/NLD/PLA1* can increase the HIR to ~4% (Li *et al*., 2021). ROS has long been recognized as an underlying cause of reproductive disorders in humans and plants. In fact, ROS‐mediated DNA fragmentation and limited sperm motility are two of the main reasons for male infertility in humans (de Ligny *et al*., 2022; Tremellen, 2008). In plants, while the sperm nuclei with fragmented DNA normally complete fertilization, paternal genetic material will be degraded after fertilization, and the haploid embryo will ultimately form (Figure 1C and Figure 2).

MTL/NLD/PLA1 protein is conserved across monocots. Knocking out *OsMATL*, the rice (*Oryza sativa*) homologue of *ZmMTL*, decreased seed setting and generated 2–6% haploids in *indica* rice cultivars (Yao *et al*., 2018). Similarly, a HI line was developed in foxtail millet (*Setaria italica*) by knocking out *SiMTL* using CRISPR/Cas9 technology, achieving a haploid frequency of approximately 2.7% in inbred offspring (Cheng *et al*., 2021). The average HIR of *Hvmtl* mutants in barley (*Hordeum vulgare*) reached 12.5% and 11.1% following selfing and outcrossing, respectively (Tang *et al*., 2023). The gene‐edited loss‐of‐function mutant *scmtl* in sugarcane (*Saccharum spontaneum*) resulted in a HIR of 0.59%–0.96% in hybrid offspring (Guo *et al*., 2024). Application of the gene‐edited *TaPLA* in wheat (*Triticum aestivum*) produced 2–3% haploids, while not affecting the growth or development of the wheat plant or pollen vitality (Liu *et al*., 2020a; Sun *et al*., 2022). Using primary transformants harbouring an edited *TaMTL*, an HI rate of 18.9% was obtained in T1 plants (Liu *et al*., 2020b). The successful identification of sugarcane and wheat haploids indicates that the HI technology based on the *MTL/NLD/PLA1* gene is not restricted to diploid plants.

Loss‐of‐function mutants of the gynoecium‐expressed *phospholipase AII* (*pPLAIIγ*) induced on average 1.07% haploid progenies in *Arabidopsis thaliana* (Jang *et al*., 2023). However, in contrast to the function of *MTL/NLD/PLA1* in monocots, the loss of function of the Arabidopsis *pPLAIIγ* gene resulted in the generation of maternal haploid plants (Gilles *et al*., 2017a; Jang *et al*., 2023; Kelliher *et al*., 2017; Liu *et al*., 2017). Notably, haploid offspring were derived from the maternal genome and initiated by the female side rather than pollen (Jang *et al*., 2023). This finding suggests that the mechanism by which phospholipases mediate haploid induction differs between *PLA1* in monocots and *pPLAIIγ* in Arabidopsis, highlighting the need for further investigation into the potential application of *PLA1* in dicot species.

Based on these genes, inducer lines have been obtained in maize and other crops by specifically inducing the degradation of the genetic material from the male parent. Unfortunately, inducer lines based on DNA fragmentation sometimes face genotype‐specific limitations, underscoring the need to develop additional HI methods. A non‐genetic approach based on treating pollen with a variety of chemicals, such as phosphatidylcholine and methimazole, raises ROS levels and induces haploids, thus providing alternative non‐*genetically modified organism (GMO)* methods for breeders (Figure 2) (Jiang *et al*., 2022; Ruban and Houben, 2022). We anticipate that additional gene‐ or chemical‐based techniques to increase ROS levels will be identified, and their application in breeding will widen.

## Disruption of plasma membrane communication and fusion (H2)

The fusion of the plasma membrane in double fertilization is a complex process (Figure 1D). This fusion proceeds through adhesion, recognition and insertion and is directly regulated by multiple proteins on the surface of each gamete membrane (Dresselhaus *et al*., 2016). Among these regulators, egg cell‐secreted small cysteine‐rich EGG CELL 1 (EC1) provides the fusion competence for sperm cell adhesion and separation (Sprunck *et al*., 2012). On the male gamete side, the membrane proteins HAPLESS 2 (HAP2, also reported as GENERATIVE CELL SPECIFIC 1 [GCS1]) and GAMETE EXPRESSED 2 (GEX2) regulate the adhesion and fusion of plasma membranes from each gamete (Mori *et al*., 2014, 2006; Zhang *et al*., 2021). DOMAIN OF UNKNOWN FUNCTION 679 (DUF679) protein 9 (DMP9) is a small transmembrane protein of 244 amino acids whose encoding gene is co‐expressed with the sperm cell‐specific gene *GEX2* (Cyprys *et al*., 2019).

A single‐nucleotide polymorphism in the pollen‐expressed gene *DMP*, leading to a single amino acid change in the first predicted transmembrane domain, was found to be the causative reason behind the quantitative trait locus *qhir8* associated with haploid induction in maize (Zhong *et al*., 2019). Since then, *DMP* genes have been utilized for HI in numerous plant species (Liu *et al*., 2015; Zhong *et al*., 2019). Maternal haploids can be induced by loss‐of‐function mutations in the *ZmDMP‐like* Arabidopsis genes *DMP8* and *DMP9*, with an average HIR of 2.1% ± 1.1% (Zhong *et al*., 2020). Moreover, *AtDMP8* and *AtDMP9* exhibit similar expression patterns to *ZmDMP*, with comparatively elevated expression in tissues such as pollen, stamens and flower buds (Zhong *et al*., 2019). Consistent with their function in HI in maize, manipulating *DMP* function can also trigger *in vivo* maternal HI in barrel clover (*Medicago truncatula*, HIR ranged from 0.29%–0.82%), rapeseed (*Brassica napus*, HIR of 2.4%), cabbage (*Brassica oleracea*, HIR ranged from 0.41%–2.35%), tobacco (*Nicotiana tabacum*, HIR of 1.2%), tomato (*Solanum lycopersicum*, HIR ranged from 0.5–3.7%), potato (*Solanum tuberosum*, HIR is approximately 0.005–0.01%), watermelon (*Citrullus lanatus*, HIR ranged from 0.51%–1.12%), upland cotton (*Gossypium hirsutum*, HIR ranged from 0.3%–1.06%) and soybean (*Glycine max*, HIR ranged from 0.36%–0.76%) (Chen *et al*., 2023; Li *et al*., 2022; Long *et al*., 2023; Tian *et al*., 2023; Wang *et al*., 2022b; Zhang *et al*., 2022; Zhao *et al*., 2022b; Zhong *et al*., 2022a, 2022b, 2024). These results suggest that haploids may be induced by other types of mutants with defective male–female gamete interactions. While DMP8 and DMP9 help to form fusion‐competent sites, as well as HAP2 trafficking or activation, which all contribute to the fusion between gametes (Takahashi *et al*., 2018; Wang *et al*., 2022c). DMP8 and DMP9 are essential for the EC1‐induced translocation of HAP2/GCS1 from discrete vesicle‐like structures within sperm to the sperm plasma membrane, where HAP2/GCS1, DMP8 and DMP9 proteins then remain highly associated (Wang *et al*., 2022c). This regulatory function highlights the essential role of AtDMP8/9 in ensuring successful gamete fusion by orchestrating the localization of cell‐fusion machinery within the sperm cell membrane. These findings strongly suggest that impaired gamete fusion, resulting from defective DMP‐mediated protein trafficking, may be a key factor contributing to HI in *DMP* mutant plants (Figure 1E). However, the precise molecular mechanisms underlying *DMP*‐mediated HI remain largely unknown. Furthermore, the genetic modification of *GEX2* and *HAP2/GCS1*, which also play indispensable roles in gamete plasma membrane fusion, may provide additional avenues for HI.

**Figure 3:**
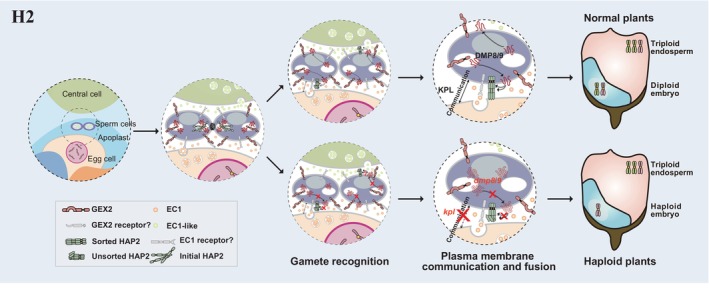
Haploid induction via disrupted communication and fusion between the plasma membranes of the male and female gametes.

*KOKOPELLI* (*KPL*) in *Arabidopsis* is a sperm‐specific natural antisense short interfering RNA (nat‐siRNA) expressed in the male gametophyte from a locus immediately upstream of an inversely transcribed gene named *ARIADNE14* (*ARI14*), encoding a potential ubiquitin E3 ligase (Ron *et al*., 2010). Loss of the KPL function leads to severe defects specifically during the double fertilization, resulting in failed fertilization events (Grant‐Downton and Rodriguez‐Enriquez, 2012). The *kpl* mutant leads to a single fertilization event and triggers in planta maternal HI (HIR ranged from 0.07%–0.34%, Figure 1E and Figure 3) (Jacquier *et al*., 2023). Understanding the complex genetic pathways controlling the activation, attachment and fusion of sperm and egg cells is essential to successfully harnessing this pathway for HI.

## Blocking nuclear membrane fusion (H3)

**Figure 4:**
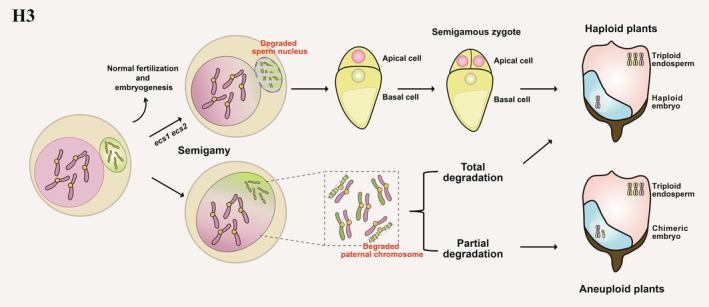
Haploid induction via blocking the fusion of the male and female nuclear membranes. Total degradation of paternal chromosomes will result in haploidy, while partial chromosome degradation will lead to the appearance of chimeric embryos.

Nuclear fusion is a crucial stage for sexual reproduction in animals, plants and fungi. During double fertilization, nuclear fusion occurs three times in flowering plants: once during female gametogenesis, when the two polar nuclei fuse to generate the central cell; and twice during double fertilization, when the sperm cells fertilize the egg cell and the central cell (Maruyama *et al*., 2010; Willemse and van Went, 1984). Nuclear fusion is achieved by the fusion of the nuclear membranes from the sperm and that of the egg nucleus, forming the zygotic membrane (Figure 1F and Figure 4) (Jensen, 1964). During nuclear membrane fusion, the two parental haploid nuclei fuse to generate one diploid nucleus (Kobayashi and Nishikawa, 2023).

Semigamy is defined as the failure of the sperm and egg nuclei to fuse properly after zygote formation during fertilization and has been observed in many plant species (Coe, 1953; Curtiss *et al*., 2012; Jia *et al*., 1989; Lanaud, 1988; Rao and Narayana, 1980; Turcotte and Feaster, 1963). The semigametic line 57–4 in *Gossypium barbadense* was isolated from a doubled haploid isogenic mutant and produces 30%–60% haploid offspring following self‐pollination (Turcotte and Feaster, 1967). Embryo observations suggest that abnormal fusion between the sperm nucleus and the egg nucleus is likely a pivotal determinant in haploid formation (Zhang *et al*., 1996). The action of the causative gene that can explain the observed semigamy in the 57–4 cotton line remains elusive, and its molecular identity has yet to be characterized and cloned. Therefore, being able to locate this gene could help to expand the HI toolbox greatly.

A recent study demonstrated that a double mutant lacking the function of EGG CELL‐SPECIFIC ASPARTIC ENDOPEPTIDASE 1 (ECS1) and ECS2 exhibits a defect in gamete fusion and produces haploid seeds that lack the fluorescence signal from a transgene harboured by the paternal genome (Figure 1G and Figure 4) (Zhang *et al*., 2023). Thus, the haploid offspring produced by *ecs1 ecs2* mutants are thought to arise from semigamy leading to maternal haploid individuals (HIR ranged from 0.0086%–0.035% and 0.8%–1.1%), which was confirmed through reciprocal crosses (Mao *et al*., 2023; Zhang *et al*., 2023). Although the sperm nucleus fails to fuse with the egg nucleus, it initiates the development of the egg cell into a zygote after the sperm nucleus enters the egg cell (Zhang *et al*., 2023). Earlier investigations determined that ECS1 and ECS2 are secreted into the extracellular space to avoid fusion with multiple pollen tubes (known as polytubey), but only after the egg cell senses successful fertilization (Yu *et al*., 2021; Zhang *et al*., 2023). Importantly, mutating the rice homologues of ECS1 and ECS2 also resulted in the isolation of haploids in selfed and hybrid progeny of *Osecs* CRISPR‐generated mutants, with HIR ranging from 0.031%–0.036% (Zhang *et al*., 2023). These findings indicate that the ECS system may share similar functions in dicot and monocot plants, thus providing insight into developing another HI system.

Previous studies indicate that *ECSs* are essential for facilitating the fusion of male and female nuclei (Zhang *et al*., 2023). Proteins involved in gamete nuclear membrane fusion regulation could be potential candidates for haploid induction via a mechanism similar to *ECS*. For instance, the yeast Ig binding protein BiP has a number of orthologs in *Arabidopsis*, where the *bip1 bip2* double mutant is defective in the fusion of polar nuclei; likewise, loss of function in any two of the BiP partners ER‐resident soluble J proteins P58IPK, ERdj3A and ERdj3B showed defects in polar nuclear fusion (Maruyama *et al*., 2010; Maruyama *et al*., 2014). Similarly, the *bip1 bip2* and *erdj3a p58ipk* double mutants were defective in fusion between the sperm nucleus and the central cell nucleus (Maruyama *et al*., 2020). The expression of BiP3 under the control of the BiP1 promoter fully rescued the defective phenotype of polar nuclear fusion in the *bip1 bip2* mutant (Maruyama *et al*., 2015). Finally, Sad1/UNC84 (SUN), GEX1 and KARyogamy 5 (Kar5) proteins carry out functions in nuclear fusion in *Arabidopsis* (Hwang *et al*., 2019; Nishikawa *et al*., 2020; Yabe and Nishikawa, 2022). These genes have been implicated in nuclear membrane fusion and may serve as candidates for HI if they can compensate for the polar nuclear fusion defect.

## Uniparental chromosome elimination (H4)

Following the fusion of the nuclear envelopes, chromosomes from the male and female gametes move under the traction of spindle poles to initiate embryogenesis (Risteski *et al*., 2021). This orchestrated mitotic movement is crucial for ensuring precise chromosome segregation (Figure 1H) (Tiang *et al*., 2012). The centromere is a specialized chromosome region responsible for the attachment of microtubules from the spindle apparatus to mediate proper separation of sister chromatids. CENTROMERIC HISTONE 3 (CENH3, also reported as centromere protein A [CENP‐A]) is a specific variant of histone H3, replacing canonical histone H3 and maintaining the structural integrity and function of the centromere (Müller and Almouzni, 2017). CENH3 contains a highly conserved DNA‐binding histone‐fold domain that forms the specific chromosome centromeric positioning regions, together with a variable N‐terminal tail domain that interacts with kinetochore proteins (Malik and Henikoff, 2003). It is mainly involved in the recruitment and stability of the kinetochore complex, which is necessary for chromosome separation and plays a vital role in defining centromere positions on chromosomes (Allshire and Karpen, 2008). Any error in the transcription of *CENH3* or translation, modification or centromere incorporation of CENH3 can disrupt the interaction between the centromere and the mitotic spindle, which can lead to selective centromere dysfunction and chromosome loss (Allshire and Karpen, 2008).

**Figure 5:**
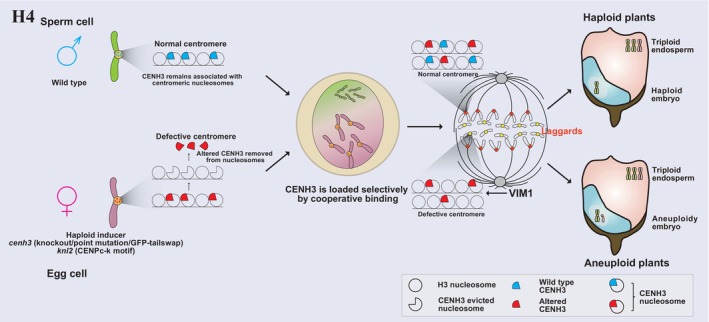
Haploid induction via the CENH3‐HI system leading to uniparental chromosome elimination. CENH3 function is directly (*cenh3*) or indirectly (*knl2*) disturbed, causing the elimination of maternal or paternal chromosomes during early embryogenesis.

A groundbreaking publication reported that transgenic *Arabidopsis* plants expressing a modified CENH3 variant induced haploids when crossed to the wild type (Ravi and Chan, 2010). The N‐terminal tail of histone H3.3 was fused to the green fluorescent protein (GFP), and the encoding construct was used to complement a *cenh3* knockout mutant; the resulting line was designated GFP‐tailswap (Ravi and Chan, 2010). Intriguingly, a high rate of haploid and aneuploid progeny (25%–45% of viable offspring) was obtained when GFP‐tailswap plants were used as female parents for a cross to wild‐type plants (Ravi and Chan, 2010). Another approach involved adding the GFP‐tag to the N‐terminal of wild‐type CENH3 (GFP‐CENH3), which also induced haploid formation (5%) when a female GFP‐*CENH3* transgenic plant was crossed to wild type (Ravi *et al*., 2011). A distinctive feature of this CENH3‐mediated HI is its efficiency in generating haploid seeds; using the inducer line (GFP‐tailswap or GFP‐*CENH3* mutant) as the female or male parent (Ravi *et al*., 2011; Ravi and Chan, 2010). Subsequent studies have shown that point mutations in the CENH3 α‐N‐helix or centromere‐targeting domain (CATD), as well as the replacement of *Arabidopsis* CENH3 with orthologs from related plant species, can also induce haploids (Karimi‐Ashtiyani *et al*., 2015; Kuppu *et al*., 2015; Maheshwari *et al*., 2015). Importantly, CENH3 point mutations may be generated by a non‐transgenic method like targeting‐induced local lesions in genomes (TILLING) using chemical mutagenesis. The introduction of in‐frame deletions via CRISPR‐mediated gene editing produced haploid progeny at a frequency of up to 25.7% (Kuppu *et al*., 2020). In addition, an enhanced yellow fluorescent protein (EYFP)‐ tagged CENH3 derived from a transgene in the *cenh3* mutant background in combination with an anti‐GFP nanobody targeted the ubiquitin proteasome system to degradate the tagged CENH3 protein, offering an additional approach for haploid induction in *Arabidopsis* (Demidov *et al*., 2022). The centromere handicap driven by direct or indirect modifications of the CENH3 protein will evoke uniparental chromosome elimination and then induce haploid (Figure 5) (Hewawasam *et al*., 2010; Marimuthu *et al*., 2021; Niikura *et al*., 2019; Ranjitkar *et al*., 2010).

CENH3‐based haploid‐inducing systems have been applied for different crops, but the efficiency of this approach is much less than that obtained in *Arabidopsis*. In maize, feasible HI based on modified CENH3 has been demonstrated (Kelliher *et al*., 2016; Wang *et al*., 2021). The HIR of the AcGREEN‐tailswap‐CENH3 transgene in the *cenh3*/*cenh3* background reached a maximum of 3.6% when stated on the paternal side of the cross (Kelliher *et al*., 2016). Pollinating *cenh3*/+ maize plants with pollen from *glossy1* (*gl1*) or *gl8* mutants resulted in haploid frequencies of approximately 5% in the progeny, while using *cenh3*/+ as a male parent triggered 0.5% HIR (Wang *et al*., 2021). A paternal HI line in wheat was identified by screening genome‐edited TaCENH3α‐heteroallelic combinations that achieved approximately 7% HIR, whereas the direct transmission of the null *cenh3* allele failed to induce haploids (Lv *et al*., 2020). A recent study reported that the manipulation of CENH3 in switchgrass (*Panicum virgatum*) induces 0.5%–1.4% haploids (Yoon *et al*., 2023). Furthermore, CENH3‐based HI systems of low efficiency were reported for tomato (*Solanum lycopersicum*), rice (*Oryza sativa*), cucumber (*Cucumis sativus*), melon (*Cucumis melo*), onion (*Allium cepa*), cauliflower (*Brassica oleracea* var. *botrytis*), kale (*Brassica oleracea* var. *sabellica*) and broccoli (*Brassica oleracea* var. *italica*) (Han *et al*., 2024; Lv *et al*., 2020; Lv and Kelliher, 2023; Manape *et al*., 2024; Maria *et al*., 2017; Rik *et al*., 2017; Wang *et al*., 2024a). The role of CENH3 in soybean has been investigated, and although knockout of CENH3 did not lead to haploid production, aneuploids have been reported (Wang *et al*., 2023a). These reports suggest the potential application for plants with a modified CENH3 as haploid inducers, but further optimization is required.

Multiple factors influence the HIR frequency of crosses involving a modified CENH3. The *Arabidopsis* accession Bor‐4 carries a loss‐of‐function allele for the E3 ubiquitin ligase VARIANT IN METHYLATION 1 (VIM1), a methyl cytosine binding protein, leading to hypomethylated centromeric repeats, decondensed centromeres and decreased CENH3 abundance (Johnson *et al*., 2007; Kraft *et al*., 2008; Woo *et al*., 2007). Crossing *cenh3* GFP‐tailswap to the Bor‐4 accession increased HIR to 70% (Marimuthu *et al*., 2021). The null allele *vim1‐2* in the wild‐type Col‐0 background also improved the HIR when used as a female or male parent (Marimuthu *et al*., 2021). The challenges in establishing the Arabidopsis‐based HI system in crops may stem from species‐specific differences in the quantitative or developmental regulation of *CENH3* deposition and stability (Lv *et al*., 2020; Wang *et al*., 2021). Notably, while the GFP‐tailswap modification is viable in *A. thaliana*, it proves to be lethal in *Zea mays* (Wang *et al*., 2021). Previous studies have suggested that optimizing HI efficiency across different plant species may necessitate species‐specific modifications of CENH3 (Marimuthu *et al*., 2021).

Combining the *cenh3‐1* GFP‐tailswap HI system and a mutation in the DNA ligase IV gene *LIG4* also increased HIR (Tan *et al*., 2015). *LIG4* is a highly conserved component of the traditional non‐homologous end joining pathway in eukaryotes (Tan *et al*., 2015). Likewise, changing the growth temperature affects the efficiency of HI by the CENH3‐based HI systems. Indeed, higher temperatures raise HI efficiency with diminished pollen viability, while lower temperatures lead to the opposite effect (Ahmadli *et al*., 2023; Jin *et al*., 2023; Wang *et al*., 2023b). These findings describe a simple and effective method for improving CENH3‐based haploidization in plants.

*KINETOCHORE NULL2* (*KNL2*, also reported as *Mis18‐binding protein 1* [*M18BP1*]), a CENH3 assembly factor, is involved in regulating the loading and deposition of CENH3 onto centromeres (Boudichevskaia *et al*., 2019; Lermontova *et al*., 2013; Moree *et al*., 2011). KNL2 contains a conserved CENPC‐like motif, designated CENPC‐k, at its C terminus that is required for the centromeric localization of KNL2 *in vivo* (Sandmann *et al*., 2017; Zuo *et al*., 2022). Mutating conserved amino acids within the CENPC‐k motif or complete deletion of the motif abolishes the centromeric localization of KNL2 (Sandmann *et al*., 2017). Knocking out *KNL2* in Arabidopsis and using the mutant as a female in a cross produced a 10% rate of paternal HI under high temperature (Figure 1I and Figure 5) (Ahmadli *et al*., 2023). A comprehensive exploration into the distinctive roles of various domains within the KNL2 protein family promises to unveil the intricate machinery governing CENH3 loading, shedding new light on the precise mechanisms underlying centromere localization and its crucial interactions with microtubules during cell division. The resulting insights hold the potential to advance our understanding of centromere biology, with implications for innovative approaches in HI techniques.

## Ectopic expression of the embryogenesis activator (H5)

**Figure 6:**
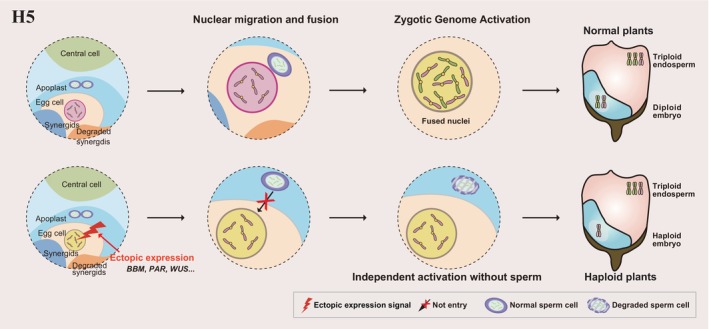
Haploid induction via the ectopic expression of *BBM*, *PAR*, or *WUS*, leading to advanced independent activation of egg cells to generate haploids without the paternal genome.

The completion of the double fertilization is marked by signalling leading to maternal‐to‐zygotic transition and zygotic genome activation (Figure 1J and Figure 6). At the end of fertilization, maternal and paternal transcripts come together in the cytoplasm of the zygote due to plasmogamy; the mRNAs such as *BABY BOOM* (*BBM*), delivered by the sperm nucleus into the zygote, have an important role in the transition or activation step (Dresselhaus and Jürgens, 2021). However, the premature initiation of this signal in egg cells might lead to the direct development of haploid embryos without the prior fusion of sperm nuclei. The egg cell therefore remains unfertilized, although the central cell's fertilization takes place, resulting in normal endosperm development and the production of viable haploid embryos.

*BABY BOOM (BBM)*, an *AINTEGUMENTA‐like* transcription factor gene, belongs to the *APETALA2*/*ETHYLENE RESPONSE FACTOR* (*AP2*/*ERF*) family, which was shown to play a critical role in inducing or enhancing somatic embryogenesis (Boutilier *et al*., 2002; Conner *et al*., 2015; Jha and Kumar, 2018; Khanday *et al*., 2019; Lutz *et al*., 2015). In addition to triggering somatic embryogenesis, recent studies have demonstrated that the ectopic expression of *BBM* in egg cells can trigger egg cells to initiate embryogenesis in flowering plants (Figure 1K and Figure 6) (Chen *et al*., 2022; Conner *et al*., 2015; Khanday *et al*., 2019). Genetic engineering approaches using transgenes driving the expression of *PsASGR‐BABY BOOM*‐*like* (*PsASGR*‐*BBML*) from fountain grass (*Pennisetum squamulatum*) or *OsBBM1* have successfully induced parthenogenesis in rice, maize and pearl millet (*Pennisetum glaucum*) (Chahal *et al*., 2022; Conner *et al*., 2015, 2017; Khanday *et al*., 2019; Skinner *et al*., 2023). These transgenes, when expressed under specific promoters, can stimulate parthenogenesis and the production of viable haploid seeds by parthenogenesis. Recently, the ectopic expression of *BBM* in Arabidopsis, *B. napus*, tomato and maize egg cells successfully bypassed fertilization for embryogenesis (Chen *et al*., 2022; Skinner *et al*., 2023). The *BBM* transcriptional network governing embryo identity and development directly modulates the expression of *LEAFY COTYLEDON1* (*LEC1*), *LEC2*, *ABI3* and *FUSCA3* (Boutilier *et al*., 2002; Braybrook and Harada, 2008; Gazzarrini *et al*., 2004; Parcy *et al*., 1994; Stone *et al*., 2008; Tao *et al*., 2017; Tian *et al*., 2020). Deciphering the intricate regulatory network of BBM also contributes to a better understanding of its role in triggering HI.

The *PARTHENOGENESIS* (*PAR*) gene was recently isolated in apomictic dandelion (*Taraxacum officinale*). This gene encodes a zinc finger protein with an ethylene‐responsive element‐binding factor‐associated amphiphilic repression (EAR) domain (Figure 1K and Figure 6) (Underwood *et al*., 2022). The ectopic expression of *PAR*, under the control of the *Arabidopsis* EC1 promoter, triggers the development of haploid embryos within unfertilized embryo sacs when introduced into sexual lettuce (*Lactuca sativa*) (Underwood *et al*., 2022). The recessive *par* gene is active in the pollen of sexual dandelion. Additionally, two *PAR* homologues in *Arabidopsis*, namely, *DUO1‐ACTIVATED ZINC FINGER 3* (*DAZ3*) and *TRANSCRIPTIONAL REPRESSOR OF EIN3‐DEPENDENT ETHYLENE‐RESPONSE 1* (*TREE1*), rank among the most highly expressed genes in *Arabidopsis* sperm cells (Underwood *et al*., 2022). Consequently, the role of the *PAR* gene was proposed to alleviate the intrinsic inhibition of embryogenesis in unfertilized egg cells (Underwood *et al*., 2022). This study underscores the potential application of *PAR* homologues for inducing parthenogenesis in other dicot species. In addition, the *proAtDD45*:*ToPAR* transgene consisting of the *Arabidopsis DD45* promoter driving expression of *PAR* from dandelion can also induce haploid production (higher maximum HIR up to 10.2%) in foxtail millet (Huang *et al*., 2024).

Apart from *BBMs* and *PARs*, the gene *RWP*‐*RK DOMAIN*‐*CONTAINING 4* (*RKD4*, also reported as *GROUNDED* [*GRD*]) was identified, triggering gene expression and pattern formation during early embryogenesis in *Arabidopsis* (Waki *et al*., 2011). The locus containing the *RWP* gene was also shown to be associated with apomixis in apomictic *Fortunella* and *Citrus* (Shimada *et al*., 2018; Wang *et al*., 2022a). Another member of the *RKD* family, *AtRKD5*, inhibits *AtBBM*‐mediated parthenogenetic potential (Liu *et al*., 2024). Ectopic expression of *AtBBM* in the egg cells was successfully enhanced in the *atrkd5* mutant, leading to the production of haploid progeny through parthenogenesis at a frequency of 0.28% (Liu *et al*., 2024). In addition, numerous plant taxa exhibit parthenogenesis, wherein embryos develop spontaneously without fertilization, resulting in the production of haploid or diploid progeny (Xiong *et al*., 2023). The *haploid initiator* (*hap*) gene in barley, which is identical to the *indeterminate gametophyte* (*ig*) mutant, can trigger the development of parthenogenic haploids (Evans, 2007; Mogensen, 1982). Recent studies have demonstrated that the ectopic expression of *OsWUSCHEL* driven by the *AtDD45* promoter can effectively induce synthetic apomixis (Huang *et al*., 2025).

Exploring the genetic characteristics and molecular mechanism of apomixis is vital for elucidating the theoretical basis of haploid breeding, yet research on apomixis is slow owing to its complexity. As the normal double fertilization of egg cells will not directly develop into zygotes autonomously without stimulation from sperm nuclei, the identification of the sperm nuclei‐specific signals that activate egg cells to initiate embryo development is of paramount importance for haploid induction studies. Indeed, the gene encoding the onset of zygotic genome activation comes from the paternal transcript and presents a distinct transcriptional activity (Anderson *et al*., 2017; Zhao *et al*., 2019). Accordingly, identifying paternally specific transcription factors that trigger zygote initiation and directly inducing the egg‐to‐zygote transition without sperm involvement could be a feasible strategy for haploid induction. Apomixis cannot occur independently of the double fertilization process. While the embryo develops without the paternal chromosome set, the proliferation of the endosperm still requires the successful fusion of the central and sperm cells.

## Expansive applications of gene editing in haploid induction

Genome editing (GE) technologies allow for precise modifications to an organism's genome, enabling the enhancement of specific traits and the in‐depth study of gene functions, providing unprecedented opportunities to develop crops with improved characteristics and to deepen our understanding of plant biology at the genetic level (Bak *et al*., 2018; Gaj *et al*., 2013; Liu *et al*., 2022; Manghwar *et al*., 2019). Currently, genome editing is being utilized to develop haploid inducer systems, enabling the broader application of haploid technology across various plant species (Delzer *et al*., 2024; Kelliher *et al*., 2019; Lv and Kelliher, 2023). Importantly, precise editing of plant genes related to double fertilization by genetic engineering may offer a convenient means to achieve rapid and simple induced haploids.

Driven by the development of genome editing, many HI genes like *MTL*/*NLD*/*PLA1* and *DMP* have been validated and applied in various plant species (Gilles *et al*., 2017a; Kelliher *et al*., 2017; Liu *et al*., 2017; Yao *et al*., 2018; Zhong *et al*., 2020, 2019). Haploid Inducer‐Edit (HI‐Edit) is a revolutionary technology for combining HI with CRISPR‐Cas9‐mediated gene editing. Crossing the elite line with a HI line carrying a gene‐editing transgene can produce an edited haploid plant containing only the non‐inducer genome (Budhagatapalli *et al*., 2020; Delzer *et al*., 2024; Kelliher *et al*., 2019) and a related method known as haploid inducer‐mediated genome editing (IMGE) (Wang *et al*., 2019a).

The artificial induction of apomixis combining CRISPR in rice provides an effective approach for producing double haploid progeny (Carman *et al*., 2011; Goeckeritz *et al*., 2024; Li *et al*., 2023; Liu *et al*., 2023, 2014; Xu *et al*., 2022). Numerous studies have explored the genetic engineering of gametophytic apomixis (Conner *et al*., 2015, 2017; Li *et al*., 2023; Mahlandt *et al*., 2023; Wang *et al*., 2024b; Wang and Underwood, 2023). Notably, the MiMe (Mitosis‐instead‐of‐Meiosis) system was initially established in Arabidopsis through the concurrent mutation of three pivotal meiotic genes, namely *SPORULATION 11*–*1* (*SPO11‐1*), *REC8* and *OMISSION OF SECOND DIVISION 1* (*OSD1*) (d'Erfurth *et al*., 2009; Fayos *et al*., 2019; Underwood and Mercier, 2022). The skipping of both meiotic recombination and diploid embryo development by combining CENH3‐mediated genome elimination (GEM) via MiMe was first reported in *Arabidopsis* as a means of generating clonal seeds (Marimuthu *et al*., 2011). Furthermore, MiMe can result in the production of clonal progeny in hybrid rice that preserve genome‐wide parental heterozygosity when it is coupled with the expression of a parthenogenesis‐related gene, such as *BBM1* or *PAR*, in the egg cell (Song *et al*., 2023; Wei *et al*., 2023; Worthington *et al*., 2019; Zhang *et al*., 2020). The same effect could be achieved by integrating the MiMe system with the knockout of *MTL* (Wang *et al*., 2019b). The utilization of synthetic apomixis in economically significant crops outside of rice remains to be established. A recent study has shown that the similarity of *OSD1* and *REC8* between *Arabidopsis* and *G. hirsutum* does not exceed 56% and *REC8* exists as two homologous pairs corresponding to the At and Dt subgenomes in cotton (Qian *et al*., 2024). Even with the identification of these three meiosis‐related homologues, the relatively low sequence similarity and limited functional characterization in different plants present significant challenges for the practical implementation of the MiMe system.

It is worth highlighting the presence of orthologs to genes like *BBM*, *PAR*, *MTL* and *DMP* and the conservation of essential meiotic genes, as discussed in previous reports (Hyde *et al*., 2023; Ma *et al*., 2018). These conserved genetic elements hold promise for future research and implementation in angiosperms. Specifically, access to more effective HI genes to combine with the MiMe system by genetic engineering strategy (genome editing or ectopic expression) stable heterosis has brought us a better vision of haploid breeding applications, making it straightforward to fix excellent agronomic traits from the F_1_ hybrids (Wang *et al*., 2024b).

## Concluding remarks and future perspectives

Haploid induction is an accidental trait resulting from perturbation of the double fertilization, characterized by the normal development of the endosperm and a zygote containing a uniparental chromosome complement. The high coordination of signal transduction between various types of cells/organelles is an essential prerequisite for successful fertilization, and defects in the gene expression profile specific to any of these steps will result in fertilization failure. This review explored five possible paths that may lead to haploid induction. Anchored in our understanding of the mechanisms of double fertilization, we hoped to provide meaningful insights regarding plant haploid induction. Strikingly, successful haploid induction in sexually reproducing plants requires the essential condition: ensuring the initiation of the natural double fertilization process and proper endosperm development, as well as eliminating the uniparental chromosome in the zygote.

The ROS burst‐triggered DNA fragmentation and degradation of sperm nuclei have been extensively investigated and adapted for their application for haploid induction (Jiang *et al*., 2022; Li *et al*., 2017; Liu *et al*., 2017; Sun *et al*., 2022). DNA fragmentation in sperm cells does not directly cause fertilization failure, but it enables the sperm to successfully pass through the recognition barriers of the egg cell plasma and nuclear membrane, thus participating in the normal fertilization process (Figure 2). Currently, this method has yet to be widely applied to haploid induction in dicots. Although the homologue of the *ZmMTL* has been identified in Arabidopsis and also contributes to haploid induction, its mechanism is not entirely identical, particularly in the transition from a paternal haploid induction system to a maternal one (Jang *et al*., 2023). For this strategy, it is essential to maintain the proper function and recognition process of the sperm and egg plasma and nuclear membranes during fertilization, while ensuring the elimination of the fragmented parental chromosome set during embryo development. Therefore, it is crucial to explore the similarities and differences in the mechanisms by which ROS‐induced DNA fragmentation affects sperm and egg cells, and more efforts are needed to explore cases of ROS level changes leading to haploid induction in dicots. Nevertheless, critical concerns still need to be further validated: 1. Are the fragmentation degrees of the two sperm cells from the same pollen tube consistent? 2. If the fragmentation extent of the two sperm cells is equal, why does the central cell develop normally into an endosperm? Is the central cell more tolerant of fragmentation? 3. If the fragmentation extent of the two sperm cells in the pollen tube is not equivalent, is there an independent option or prioritization between fusion with the central cell or egg cell?

The prerequisite for the fusion of the plasma membranes from female and male gametes includes the adhesion of transmembrane proteins GEX2 and the insertion of HAP2/GCS1 (Mori *et al*., 2014; Zhang *et al*., 2021). To date, mutations in either *GEX2* or *HAP2* have not been shown to induce the production of haploid embryos. Nonetheless, DMP8/9 regulates HAP2/GCS1 protein to promote plasmalemma fusion to ensure successful double fertilization under the induction of secreted EC1 peptides and induced haploid production (Figure 3) (Sprunck *et al*., 2012; Wang *et al*., 2022c). Although *DMP* mutants have been identified as haploid inducers in several dicotyledonous plants, there have been no successful reports of manipulating *DMP* to induce haploids in other monocotyledonous plants, aside from the initial discovery in maize where *zmdmp* was shown to induce haploids (Chen *et al*., 2023; Wang *et al*., 2022b; Zhao *et al*., 2022b; Zhong *et al*., 2020, 2019, 2022b, 2024). The loss of GEX2 and HAP2 function directly leads to embryo abortion, and impairing their interacting proteins may be a potential approach for obtaining haploids. Moreover, when looking for additional proteins that participate in the same pathway as HAP2 and GEX2, these interacting proteins should be mainly transmembrane anchored, secreted proteins, or their encoding genes should be co‐expressed with *GEX2* and *HAP2*, like *DMP* (Cyprys *et al*., 2019; Wang *et al*., 2022c).

The fusion of the nuclear membrane from the female and male gametes is the last hurdle for bringing together the parental genetic materials. At this stage, the mechanism of gamete nuclear membrane fusion remains largely unknown, which presents a significant obstacle to utilizing this strategy. Semigamy can generate up to 60% haploid offspring in *G. bardadense*; identifying the genetic factors underlying this phenomenon could provide critical insights into advancing HI strategies. Haploid induction can also benefit from the genetic mapping and cloning of semigametic genes or the identification of proteins interacting with crucial nuclear membrane proteins. Recent reports offer potential insights, but defects in polar nuclear fusion remain a critical issue for haploid generation, as normal endosperm development is essential for the germination of haploid seeds (Nishikawa *et al*., 2020). Previous studies have shown that *ECS* mutations induce haploid in Arabidopsis and rice; however, this approach has not yet been applied to other crops (Mao *et al*., 2023; Zhang *et al*., 2023). Thus far, nuclear membrane fusion defects leading to haploid induction have only been reported in this single case (Mao *et al*., 2023; Zhang *et al*., 2023). Future research should focus on identifying additional nuclear membrane fusion proteins as a key breakthrough direction. Investigating the mechanisms of nuclear membrane fusion, such as using immunostaining of microtubule and centromere marker proteins to study the dynamics of nuclear membrane and genetic material fusion, represents a promising strategy. In addition, the similarities and differences between the fusion of the nuclear membrane and the plasma membrane remain unexplored until now.

Disrupting the function of centromeric histone H3 causes uniparental chromosome elimination during chromosome movement and separation prior to the first mitotic division of the unicellular embryo is a proven method that induces haploid in various plant species (Karimi‐Ashtiyani *et al*., 2015; Kelliher *et al*., 2019; Lv *et al*., 2020; Wang *et al*., 2021). The HI‐based CENH3 system mainly relies on maternal elimination and paternal chromosome elimination has only been observed in Arabidopsis and maize, with an efficiency of about 1/10 of maternal elimination (Ravi and Chan, 2010; Wang *et al*., 2021). Maternal haploids are more relevant to modern breeding, and improving the efficiency of maternal haploids is an urgent problem to be solved. A study reported that pollinating target plants with *CENH3* GFP‐tailswap pollen cultivated at lower temperatures, followed by higher temperatures for HI, can increase the maternal HIR to approximately 24.8% (Wang *et al*., 2023b). In addition, paternal haploid is important for cytoplasmic exchange transfer male sterility, but this also means that a complete paternal haploid cannot be obtained due to the presence of maternal cytoplasm (Bortiri *et al*., 2024).

CENH3 not only serves a specific role in meiosis and mitosis but also acts during multiple developmental stages. Therefore, it should be possible to avoid detrimental effects during plant development and fertility by focusing on KNL2, a kinetochore assembly factor that interacts with CENH3 for haploid induction (Zuo *et al*., 2022). This would imply that more interacting proteins with centromeric histone could be identified and then act in HI by disrupting the centromere function and activation. Additionally, targeting the insertion of tags into endogenous CENH3 through knock‐in methods followed by degradation represents a viable strategy for haploid production. The feasibility of this approach is constrained by the advancement of genome editing technologies in different plant species.

At the stages of the maternal‐to‐zygotic transition, the signal that marks the development of egg cells into zygotes can be initiated prematurely by ectopic expression of *BBM* and *PAR* genes, contributing to maternal haploid generation (Chen *et al*., 2022; Khanday *et al*., 2019; Underwood *et al*., 2022). BBM‐induced parthenogenesis achieves an efficiency of up to 65% in monocots (maize), whereas in dicots (Arabidopsis), it is as low as approximately 0.4% (Chen *et al*., 2022; Skinner *et al*., 2023). This significant disparity poses a major challenge to the broad application of strategies involving ectopic expression of egg cell activation signals for haploid induction. We speculate that this difference is primarily attributed to the distinct mechanisms of early embryogenesis between monocots and dicots (Zhao *et al*., 2017). Additionally, the expression pattern of *BBM* varies: in maize and rice, the *BBM* gene is expressed both in sperm cells prior to fertilization and in the zygote shortly after fertilization (Anderson *et al*., 2017; Chen *et al*., 2017). In contrast, *BBM* expression is absent in gametes (sperm or egg cells) and during the early stages of zygotic embryogenesis in Arabidopsis (Chen *et al*., 2022; Liu *et al*., 2024).

The coordinated use of genome editing and HI can significantly improve breeding efficiency (Delzer *et al*., 2024; Kelliher *et al*., 2019; Wang *et al*., 2019a). During haploid induction, the simultaneous action of CRISPR tools and induction factors enables the rapid and efficient transmission of edits into advanced breeding materials, thereby mitigating the delays and high costs associated with genetic introgression (Kelliher *et al*., 2019). An effective method is to develop an intelligent editing system: it can be automatically activated under specific induction conditions and can be activated according to particular signals during the haploid induction stage. Developing such systems helps reduce unnecessary editing activities, reduce the risk of non‐target editing and improve editing accuracy. For example, a CRISPR system that responds to temperature signals is designed to activate only under specific conditions in the induction environment, thereby achieving efficient and safe editing. This intelligent system can further optimize the breeding process, avoid unnecessary editing activities and ensure high‐precision editing of target genes. Furthermore, the advancement of genome editing technologies in different crops is crucial, as it forms the foundation for integrating genetic engineering with haploid induction.

Although CRISPR‐mediated haploid induction offers a rapid approach for developing homozygous lines, challenges remain for widespread application in crops: 1. Inconsistent efficiency, the success rate of CRISPR‐induced haploid induction varies significantly across species and even among different cultivars of the same species (Liu *et al*., 2021). 2. Technical barriers, efficient delivery of CRISPR components into plant cells remains a major challenge, particularly in species with rigid cell walls. While Agrobacterium‐mediated transformation and gene gun delivery are commonly used, these methods may not be universally applicable to all plant types (Mao *et al*., 2019). 3. Off‐target effects, despite its high precision, CRISPR remains susceptible to unintended mutations, which could have unpredictable effects on plant development and traits (Zhu *et al*., 2020). 4. Regulatory and public acceptance issues, genome editing technologies encounter regulatory barriers and public scepticism, which may limit the adoption of CRISPR‐based haploid induction in certain regions; future efforts should prioritize the development of non‐transgenic plants whenever possible (Ahmad *et al*., 2021; He *et al*., 2022). In summary, while CRISPR‐mediated HI holds great promise for accelerating and refining plant breeding, overcoming these challenges will be essential for its successful implementation.

Overall, the currently reported HI strategies are constrained by various limitations. Future research should focus on the following key challenges, including the difficulty of extending HI systems from model plants to crops, differences in fertilization mechanisms between monocots and dicots, the increased complexity of applying HI in polyploid species compared to diploids, and the challenges associated with elucidating the regulatory mechanisms underlying haploid induction. Integrating multiple HI strategies within an induction system may offer a promising approach to enhancing efficiency and facilitating the broader application of haploid breeding. Moreover, the decline in seed viability and fertility is an unavoidable issue in haploid induction. Recent reports indicate that induction lines generally exhibit an abortion rate exceeding 10% (e.g. *Zmpla1*, *Zmpod65*, *Zmdmp* and *Atecs1 ecs2*), while *Atdmp8 dmp9* shows an abortion rate of over 50% and the previously identified GFP‐tailswap line produces only 60%–70% viable seeds (Jiang *et al*., 2022; Liu *et al*., 2017; Mao *et al*., 2023; Ravi and Chan, 2010; Zhang *et al*., 2023; Zhong *et al*., 2020, 2019). This limitation should be carefully addressed to broaden the application of haploid breeding.

To summarize the efficiency of these five paths to haploid induction, H1 and H5 are likely more straightforward manipulation methods to induce haploidy due to their minimal requirement of changing the genes from uniparental gametes rather than mediating the recognition and identification from biparental gametes during double fertilization. More attention should be given to screening for proteins interacting with key factors required for the H2 and H4 methods. For the H3 method, the various states of gamete nuclei from different fusion events of the nuclear membrane will produce different chimaeras and parental haploids, hindering the production of viable haploids for crop breeding applications. We hope that this review will offer substantial benefits regarding knowledge of double fertilization and the expansion of the breeders' toolbox.

## Outstanding questions

Recent advances in genetic modification have significantly broadened our knowledge of HI in plants. What are the common induction mechanisms shared by various HI methods? How can our knowledge of the intricate events during double fertilization inform the identification of additional haploid‐inducing genes and their modes of action?

Do two sperms from the same male germ unit show different degrees of DNA fragmentation under the ROS stress? If the fragmentation degree of two sperm nuclei is the same, why does the central cell normally develop into an endosperm? Is the central cell more tolerant in fertilization? If the extent of DNA fragmentation differs between both sperm, is there an independent option or prioritization between fusing with the central cell or egg cells?

The key proteins HAP2/GCS1, GEX2 and DMP play crucial roles in facilitating the fusion of gamete plasma membranes during fertilization. However, do these key proteins also mediate the fusion of the nuclear membrane? Do the proteins mediating polar nuclear fusion have a similarly effect on sperm–egg nuclear membrane fusion?

Does abnormal fusion of the gamete nuclear membrane necessarily induce chimerism? Is haploid production a special consequence of chimeric zygote development?

Beyond the delivery of paternal genetic material, do sperm cells also contribute specific transcripts or other molecules that initiate and regulate early zygote development after fusion with the egg cell?

## Author contributions

All authors contributed to the preparation of the manuscript. T.L. and Y.Z. conceptualized the review outline; T.L. wrote the original draft; C.W., J.P. and T.L. prepared the figures; J.T., Y.L., J.Y., W.C., J.R., W.G., Z.A. and S.Z. provided constructive feedback for the original draft and figures; A.H., S.J. and Y.Z. reviewed and edited the manuscript.

## Conflict of interest

The authors have no interests to declare.

## Data Availability

Data sharing not applicable to this article as no datasets were generated or analysed during the current study.
